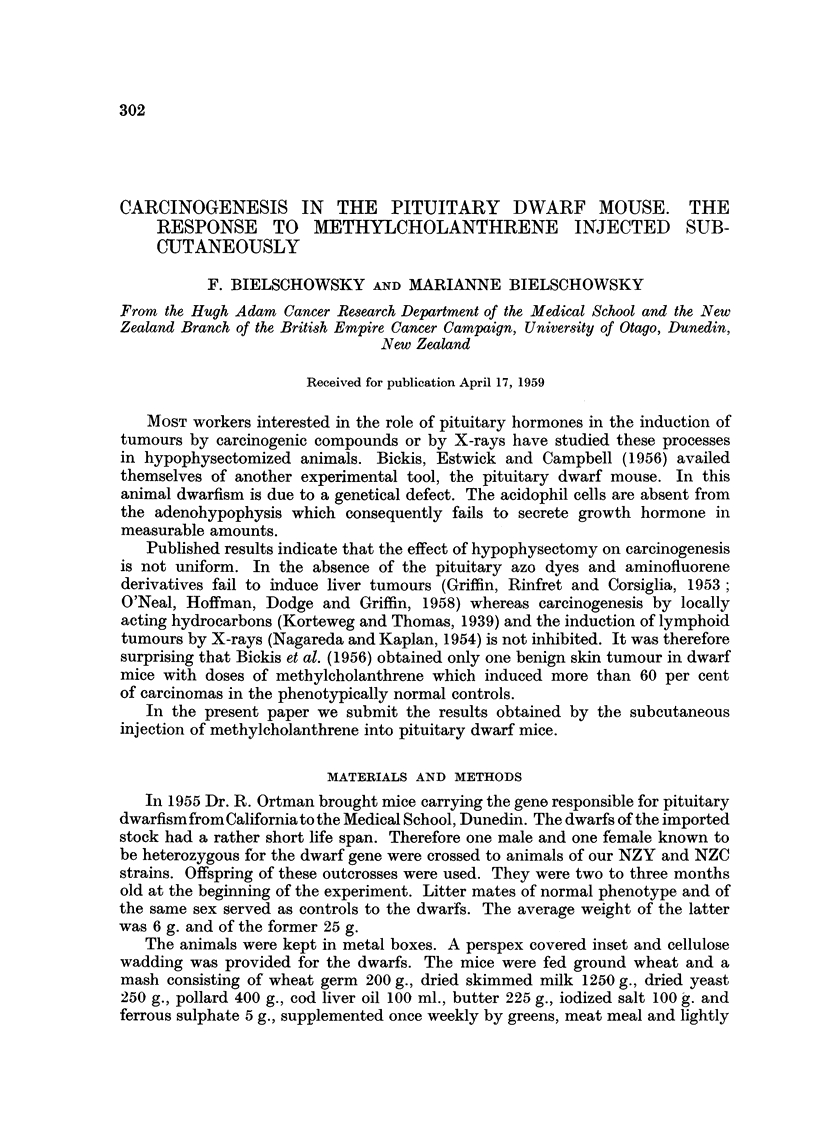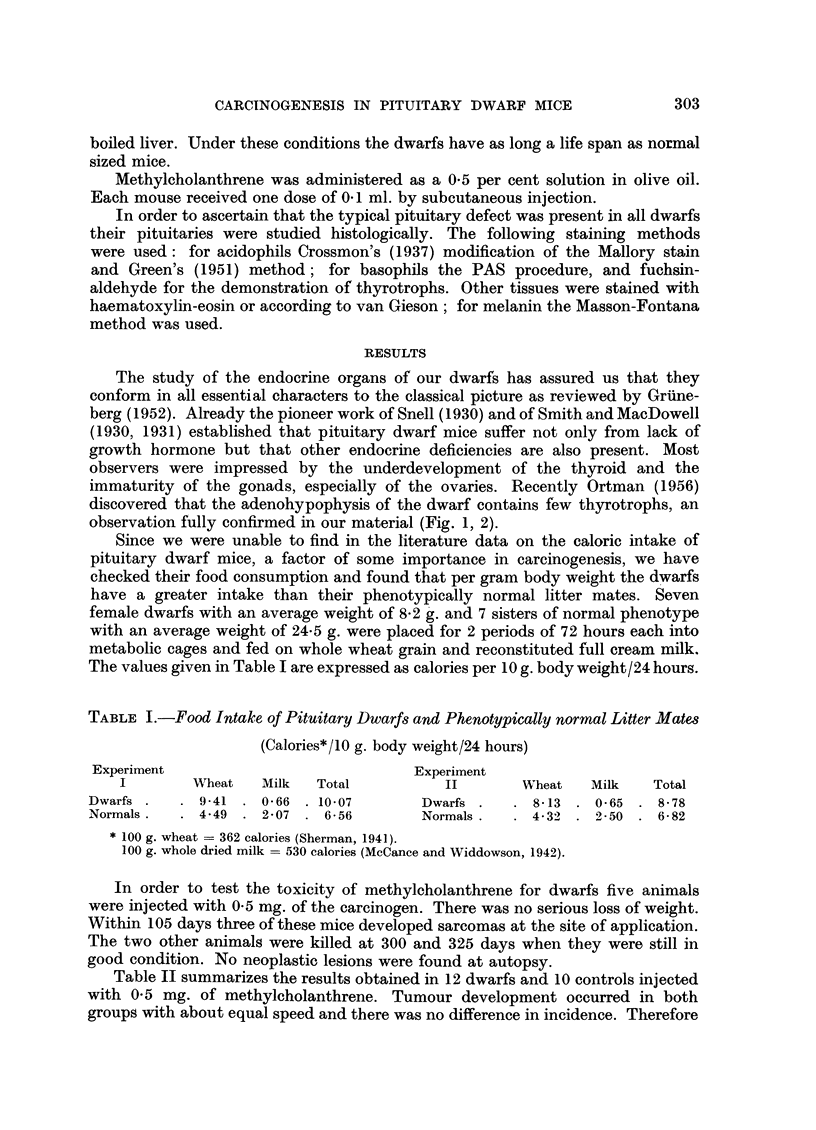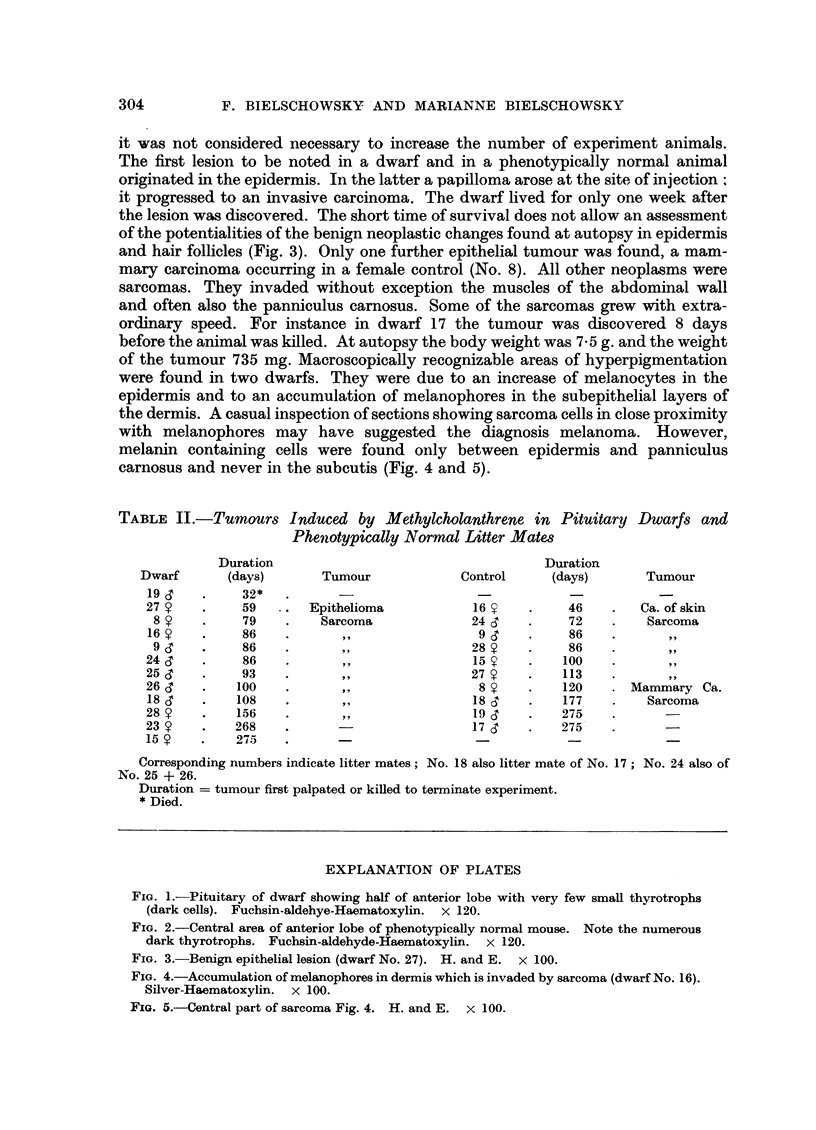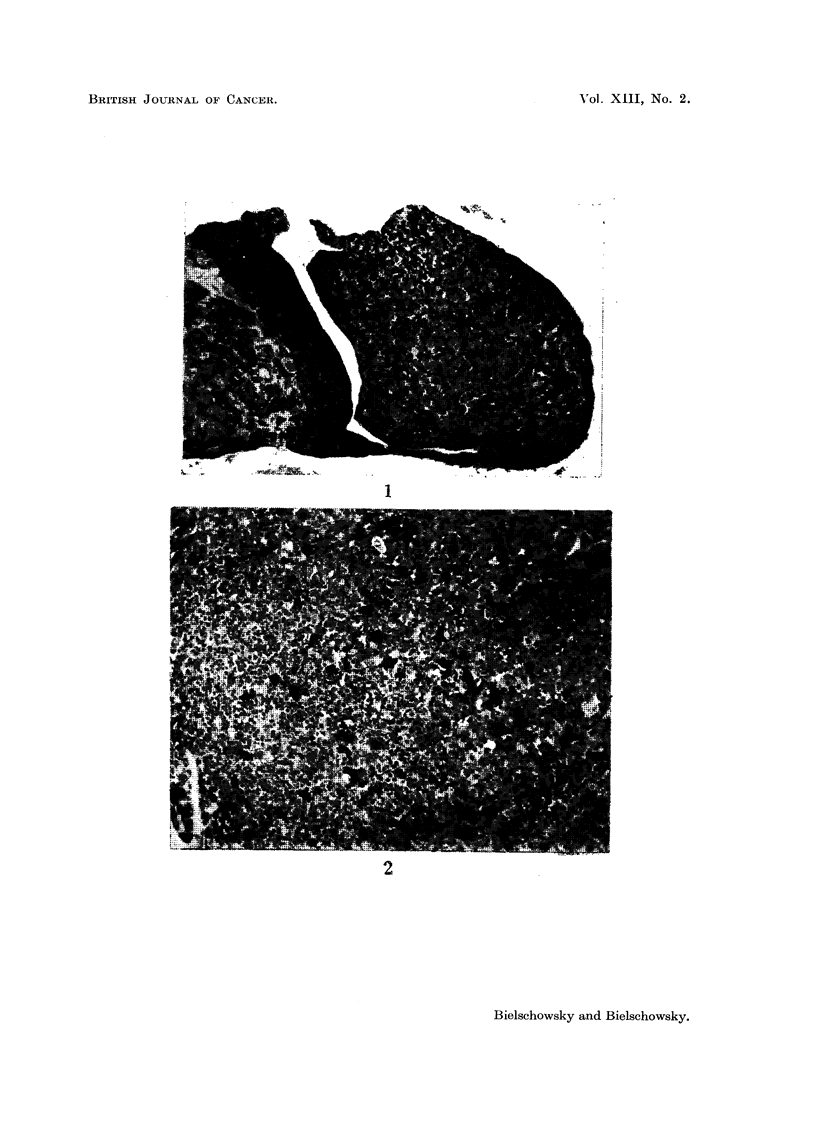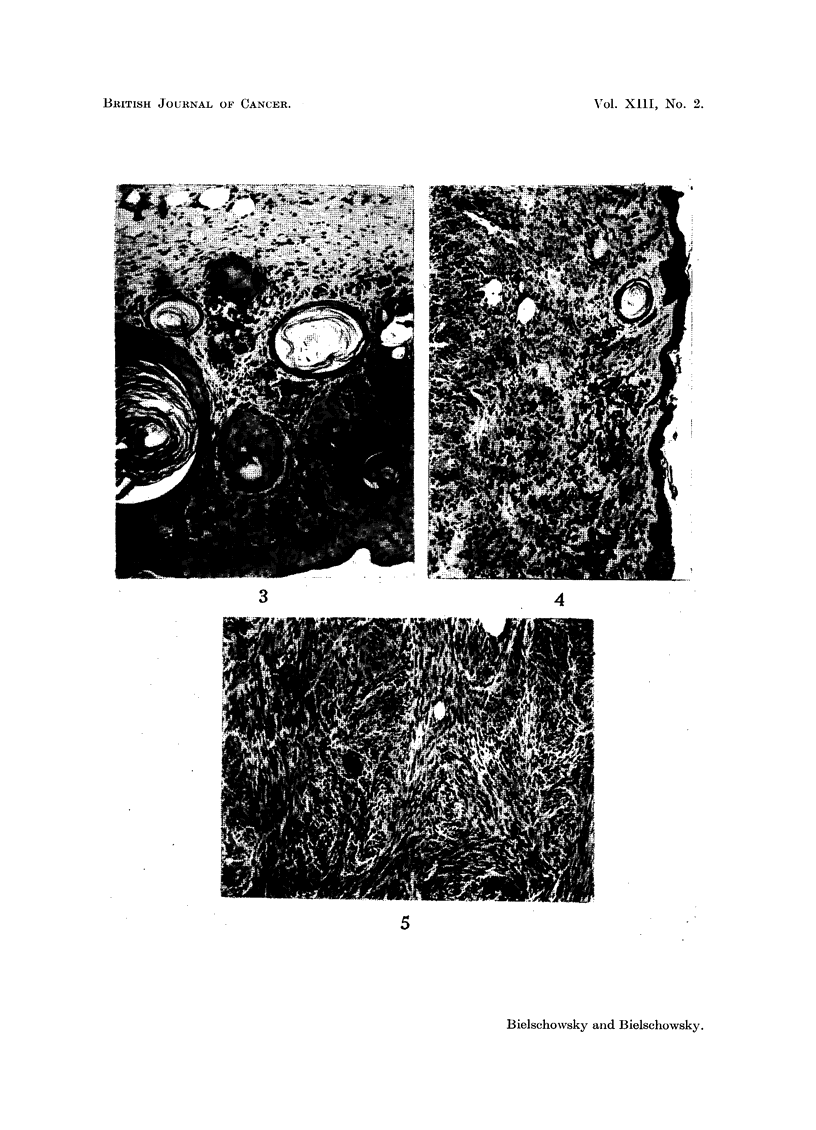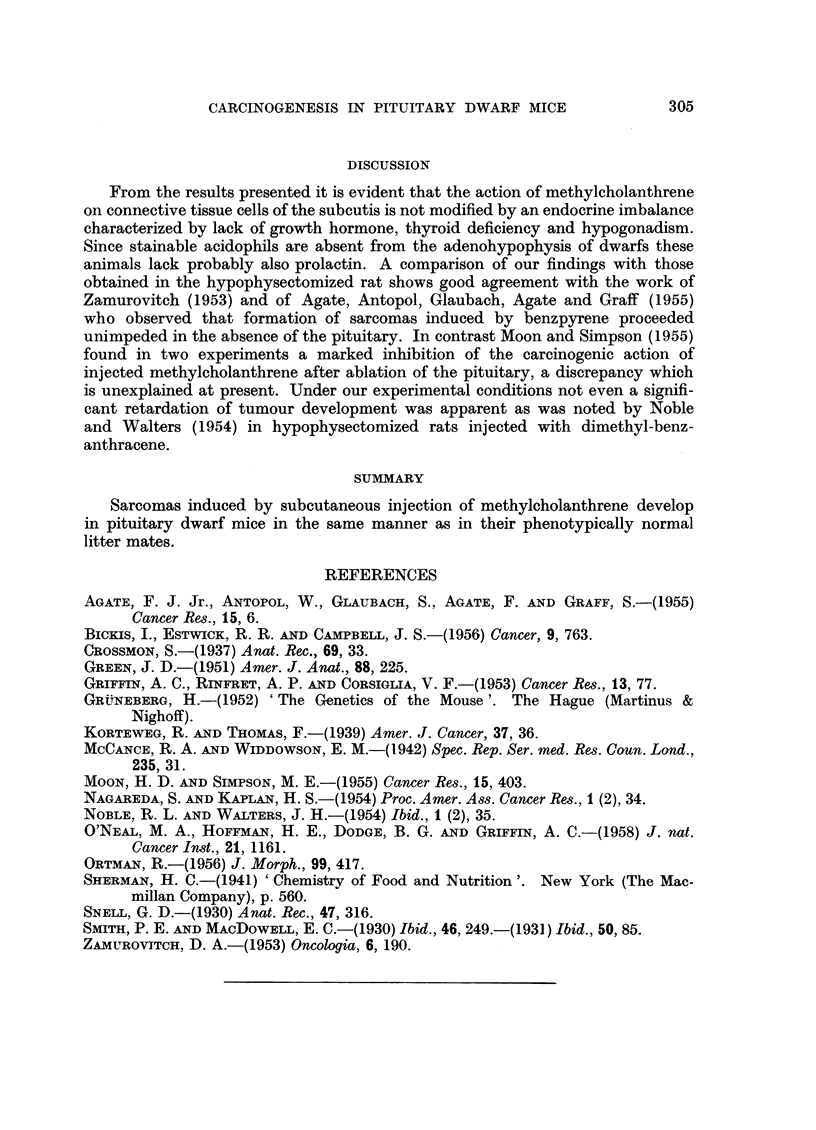# Carcinogenesis in the Pituitary Dwarf Mouse. The Response to Methylcholanthrene Injected Subcutaneously

**DOI:** 10.1038/bjc.1959.36

**Published:** 1959-06

**Authors:** F. Bielschowsky, Marianne Bielschowsky

## Abstract

**Images:**


### CARCINOGENESIS IN THE PITUITARY DWARF MOUSE. THE RESPONSE TO METHYLCHOLANTHRENE INJECTED SUBCUTANEOUSLY

F. BIELSCHOWSKY AND MARIANNE BIELSCHOWSKY

*From the Hugh Adam Cancer Research Department of the Medical School and the New Zealand Branch of the British Empire Cancer Campaign, University of Otago, Dunedin, New Zealand*

Received for publication April 17, 1959

MOST workers interested in the role of pituitary hormones in the induction of tumours by carcinogenic compounds or by X-rays have studied these processes in hypophysectomized animals. Bickis, Estwick and Campbell (1956) availed themselves of another experimental tool, the pituitary dwarf mouse. In this animal dwarfism is due to a genetical defect. The acidophil cells are absent from the adenohypophysis which consequently fails to secrete growth hormone in measurable amounts.

Published results indicate that the effect of hypophysectomy on carcinogenesis is not uniform. In the absence of the pituitary azo dyes and aminofluorene derivatives fail to induce liver tumours (Griffin, Rinfret and Corsiglia, 1953 ; O'Neal, Hoffman, Dodge and Griffin, 1958) whereas carcinogenesis by locally acting hydrocarbons (Korteweg and Thomas, 1939) and the induction of lymphoid tumours by X-rays (Nagareda and Kaplan, 1954) is not inhibited. It was therefore surprising that Bickis *et al.* (1956) obtained only one benign skin tumour in dwarf mice with doses of methylcholanthrene which induced more than 60 per cent of carcinomas in the phenotypically normal controls.

In the present paper we submit the results obtained by the subcutaneous injection of methylcholanthrene into pituitary dwarf mice.

### MATERIALS AND METHODS

In 1955 Dr. R. Ortman brought mice carrying the gene responsible for pituitary dwarfism from California to the Medical School, Dunedin. The dwarfs of the imported stock had a rather short life span. Therefore one male and one female known to be heterozygous for the dwarf gene were crossed to animals of our NZY and NZC strains. Offspring of these outcrosses were used. They were two to three months old at the beginning of the experiment. Litter mates of normal phenotype and of the same sex served as controls to the dwarfs. The average weight of the latter was 6 g. and of the former 25 g.

The animals were kept in metal boxes. A perspex covered inset and cellulose wadding was provided for the dwarfs. The mice were fed ground wheat and a mash consisting of wheat germ 200 g., dried skimmed milk 1250 g., dried yeast 250 g., pollard 400 g., cod liver oil 100 ml., butter 225 g., iodized salt 100 g. and ferrous sulphate 5 g., supplemented once weekly by greens, meat meal and lightly boiled liver. Under these conditions the dwarfs have as long a life span as normal sized mice.

Methylcholanthrene was administered as a 0·5 per cent solution in olive oil. Each mouse received one dose of 0·1 ml. by subcutaneous injection.

In order to ascertain that the typical pituitary defect was present in all dwarfs their pituitaries were studied histologically. The following staining methods were used: for acidophils Crossmon's (1937) modification of the Mallory stain and Green's (1951) method; for basophils the PAS procedure, and fuchsin-aldehyde for the demonstration of thyrotrophs. Other tissues were stained with haematoxylin-eosin or according to van Gieson; for melanin the Masson-Fontana method was used.

### RESULTS

The study of the endocrine organs of our dwarfs has assured us that they conform in all essential characters to the classical picture as reviewed by Grüneberg (1952). Already the pioneer work of Snell (1930) and of Smith and MacDowell (1930, 1931) established that pituitary dwarf mice suffer not only from lack of growth hormone but that other endocrine deficiencies are also present. Most observers were impressed by the underdevelopment of the thyroid and the immaturity of the gonads, especially of the ovaries. Recently Ortman (1956) discovered that the adenohypophysis of the dwarf contains few thyrotrophs, an observation fully confirmed in our material (Fig. 1, 2).

Since we were unable to find in the literature data on the caloric intake of pituitary dwarf mice, a factor of some importance in carcinogenesis, we have checked their food consumption and found that per gram body weight the dwarfs have a greater intake than their phenotypically normal litter mates. Seven female dwarfs with an average weight of 8·2 g. and 7 sisters of normal phenotype with an average weight of 24·5 g. were placed for 2 periods of 72 hours each into metabolic cages and fed on whole wheat grain and reconstituted full cream milk. The values given in Table I are expressed as calories per 10 g. body weight/24 hours.

TABLE I.—*Food Intake of Pituitary Dwarfs and Phenotypically normal Litter Mates*

(Calories*/10 g. body weight/24 hours)

| Experiment I | Wheat | Milk | Total | Experiment II | Wheat | Milk | Total |
|---|---|---|---|---|---|---|---|
| Dwarfs | 9·41 | 0·66 | 10·07 | Dwarfs | 8·13 | 0·65 | 8·78 |
| Normals | 4·49 | 2·07 | 6·56 | Normals | 4·32 | 2·50 | 6·82 |

* 100 g. wheat = 362 calories (Sherman, 1941).
100 g. whole dried milk = 530 calories (McCance and Widdowson, 1942).

In order to test the toxicity of methylcholanthrene for dwarfs five animals were injected with 0·5 mg. of the carcinogen. There was no serious loss of weight. Within 105 days three of these mice developed sarcomas at the site of application. The two other animals were killed at 300 and 325 days when they were still in good condition. No neoplastic lesions were found at autopsy.

Table II summarizes the results obtained in 12 dwarfs and 10 controls injected with 0·5 mg. of methylcholanthrene. Tumour development occurred in both groups with about equal speed and there was no difference in incidence. Therefore it was not considered necessary to increase the number of experiment animals. The first lesion to be noted in a dwarf and in a phenotypically normal animal originated in the epidermis. In the latter a papilloma arose at the site of injection; it progressed to an invasive carcinoma. The dwarf lived for only one week after the lesion was discovered. The short time of survival does not allow an assessment of the potentialities of the benign neoplastic changes found at autopsy in epidermis and hair follicles (Fig. 3). Only one further epithelial tumour was found, a mammary carcinoma occurring in a female control (No. 8). All other neoplasms were sarcomas. They invaded without exception the muscles of the abdominal wall and often also the panniculus carnosus. Some of the sarcomas grew with extraordinary speed. For instance in dwarf 17 the tumour was discovered 8 days before the animal was killed. At autopsy the body weight was 7·5 g. and the weight of the tumour 735 mg. Macroscopically recognizable areas of hyperpigmentation were found in two dwarfs. They were due to an increase of melanocytes in the epidermis and to an accumulation of melanophores in the subepithelial layers of the dermis. A casual inspection of sections showing sarcoma cells in close proximity with melanophores may have suggested the diagnosis melanoma. However, melanin containing cells were found only between epidermis and panniculus carnosus and never in the subcutis (Fig. 4 and 5).

TABLE II.—*Tumours Induced by Methylcholanthrene in Pituitary Dwarfs and Phenotypically Normal Litter Mates*

| Dwarf | Duration (days) | Tumour | Control | Duration (days) | Tumour |
|---|---|---|---|---|---|
| 19 ♂ | 32* | — | — | — | — |
| 27 ♀ | 59 | Epithelioma | 16 ♀ | 46 | Ca. of skin |
| 8 ♀ | 79 | Sarcoma | 24 ♂ | 72 | Sarcoma |
| 16 ♀ | 86 | ,, | 9 ♂ | 86 | ,, |
| 9 ♂ | 86 | ,, | 28 ♀ | 86 | ,, |
| 24 ♂ | 86 | ,, | 15 ♀ | 100 | ,, |
| 25 ♂ | 93 | ,, | 27 ♀ | 113 | ,, |
| 26 ♂ | 100 | ,, | 8 ♀ | 120 | Mammary Ca. |
| 18 ♂ | 108 | ,, | 18 ♂ | 177 | Sarcoma |
| 28 ♀ | 156 | ,, | 19 ♂ | 275 | — |
| 23 ♀ | 268 | — | 17 ♂ | 275 | — |
| 15 ♀ | 275 | — | — | — | — |

Corresponding numbers indicate litter mates; No. 18 also litter mate of No. 17; No. 24 also of No. 25 + 26.

Duration = tumour first palpated or killed to terminate experiment.

* Died.

### EXPLANATION OF PLATES

FIG. 1.—Pituitary of dwarf showing half of anterior lobe with very few small thyrotrophs (dark cells). Fuchsin-aldehye-Haematoxylin. × 120.

FIG. 2.—Central area of anterior lobe of phenotypically normal mouse. Note the numerous dark thyrotrophs. Fuchsin-aldehyde-Haematoxylin. × 120.

FIG. 3.—Benign epithelial lesion (dwarf No. 27). H. and E. × 100.

FIG. 4.—Accumulation of melanophores in dermis which is invaded by sarcoma (dwarf No. 16). Silver-Haematoxylin. × 100.

FIG. 5.—Central part of sarcoma Fig. 4. H. and E. × 100.

1

2

3

4

5

### DISCUSSION

From the results presented it is evident that the action of methylcholanthrene on connective tissue cells of the subcutis is not modified by an endocrine imbalance characterized by lack of growth hormone, thyroid deficiency and hypogonadism. Since stainable acidophils are absent from the adenohypophysis of dwarfs these animals lack probably also prolactin. A comparison of our findings with those obtained in the hypophysectomized rat shows good agreement with the work of Zamurovitch (1953) and of Agate, Antopol, Glaubach, Agate and Graff (1955) who observed that formation of sarcomas induced by benzpyrene proceeded unimpeded in the absence of the pituitary. In contrast Moon and Simpson (1955) found in two experiments a marked inhibition of the carcinogenic action of injected methylcholanthrene after ablation of the pituitary, a discrepancy which is unexplained at present. Under our experimental conditions not even a significant retardation of tumour development was apparent as was noted by Noble and Walters (1954) in hypophysectomized rats injected with dimethyl-benzanthracene.

### SUMMARY

Sarcomas induced by subcutaneous injection of methylcholanthrene develop in pituitary dwarf mice in the same manner as in their phenotypically normal litter mates.

### REFERENCES

Agate, F. J. Jr., Antopol, W., Glaubach, S., Agate, F. and Graff, S.—(1955) *Cancer Res.*, **15**, 6.

Bickis, I., Estwick, R. R. and Campbell, J. S.—(1956) *Cancer*, **9**, 763.

Crossmon, S.—(1937) *Anat. Rec.*, **69**, 33.

Green, J. D.—(1951) *Amer. J. Anat.*, **88**, 225.

Griffin, A. C., Rinfret, A. P. and Corsiglia, V. F.—(1953) *Cancer Res.*, **13**, 77.

Grüneberg, H.—(1952) ‘The Genetics of the Mouse’. The Hague (Martinus & Nighoff).

Korteweg, R. and Thomas, F.—(1939) *Amer. J. Cancer*, **37**, 36.

McCance, R. A. and Widdowson, E. M.—(1942) *Spec. Rep. Ser. med. Res. Coun. Lond.*, **235**, 31.

Moon, H. D. and Simpson, M. E.—(1955) *Cancer Res.*, **15**, 403.

Nagareda, S. and Kaplan, H. S.—(1954) *Proc. Amer. Ass. Cancer Res.*, **1** (2), 34.

Noble, R. L. and Walters, J. H.—(1954) *Ibid.*, **1** (2), 35.

O'Neal, M. A., Hoffman, H. E., Dodge, B. G. and Griffin, A. C.—(1958) *J. nat. Cancer Inst.*, **21**, 1161.

Ortman, R.—(1956) *J. Morph.*, **99**, 417.

Sherman, H. C.—(1941) ‘Chemistry of Food and Nutrition’. New York (The Macmillan Company), p. 560.

Snell, G. D.—(1930) *Anat. Rec.*, **47**, 316.

Smith, P. E. and MacDowell, E. C.—(1930) *Ibid.*, **46**, 249.—(1931) *Ibid.*, **50**, 85.

Zamurovitch, D. A.—(1953) *Oncologia*, **6**, 190.